# Increasing Numbers of Persons with Sleeping Problems in Sweden

**DOI:** 10.3390/diseases14010025

**Published:** 2026-01-08

**Authors:** Mona Nilsson, Lennart Hardell

**Affiliations:** 1 Swedish Radiation Protection Foundation, 178 92 Adelsö, Sweden; 2 The Environment and Cancer Research Foundation, 702 17 Örebro, Sweden; lennart.hardell@environmentandcancer.com

**Keywords:** microwaves, radiofrequency radiation, sleeping problems, Sweden

## Abstract

Objectives: This study investigated sleeping problems in the Swedish population based on the Swedish National Board of Health and Welfare’s national patient register on numbers of patients in specialized outpatient care diagnosed with codes for sleeping problems. Methods: Numbers of patients per year and per 100,000 inhabitants in various age groups: 0–4, 5–19, 20–39, 40–59, and 60+ years diagnosed each year between 2001 and 2024 with the ICD codes G47 (sleep disorders) or F51 (non-organic sleep disorders) as main diagnosis were assessed. Results: The highest increase for sleep disorders was seen among children, adolescents, and young adults. All results are given per 100,000 persons. In the age group 0–4 years, the numbers of sleep disorders (G47) increased from 41.5 in 2001 to 215.8 in 2024. The corresponding results in the age group 5–19 years were 13.8 and 235.6, respectively. In the age group 20–39 years, 40.4 were diagnosed in 2001 and 220.9 in 2024. For subjects aged 40–59 years, 169.5 were diagnosed in 2001 and 362.8 in 2024, and for persons aged 60+ years, 116.4 were diagnosed in 2001 and 322.9 in 2024. No major changes in the numbers of persons with F51, non-organic sleep disorders, were observed. Conclusions: Sleeping problems can be caused by several factors; however, the rapid increase in recent years has temporally coincided with an increase in the public’s exposure to microwave radiofrequency (RF) radiation and increasing use of screens. RF radiation and use of screens may negatively impact sleep.

## 1. Introduction

In recent decades, public exposure to microwave radiofrequency (RF) radiation has increased sharply, largely due to the widespread and increasing use of mobile phones (smartphones), along with the expansion of 3G, 4G, and, most recently, 5G network infrastructures. The proliferation of Wi-Fi routers and various other wireless devices that rely on pulse-modulated microwave signals has also contributed to this trend (see Table 3 in [1]).

Since the introduction of 5G around 2019/2020, we have published eight case reports [2,3,4,5,6,7,8,9] documenting a substantial increase in radiation levels following the rollout of 5G that exceed those previously associated with adverse health effects in people residing near mobile phone base stations [10].

### 1.1. Limits for Microwave RF Radiation

The Federal Communications Commission (FCC) in the USA [11] and the International Commission on Non-Ionizing Radiation Protection (ICNIRP) in Germany [12] have adopted or recommended exposure limits for RF radiation that are based on thermal effects (tissue heating) only. It is claimed that these limits, although only based on thermal acute effects, protect the public and workers from the negative health impacts of chronic exposure to RF radiation [13]. In 2020, the ICNIRP recommended limits that were slightly relaxed in spite of growing evidence of harmful effects below the thermal limits [12,14]. Thermal-based limits have been adopted by the majority of the countries in the world, including Sweden, although there is a group of countries that have somewhat lower limits, e.g., China, Bulgaria, Poland, Russia, and Switzerland [15].

The ICNIRP limits are important for the telecommunications and military industry sector, which is exemplified by one leading 5G infrastructure provider, as outlined in a presentation. In that presentation, it was concluded that it would be difficult or even impossible to rollout 5G with 100 times lower limits than those proposed by the ICNIRP [16].

The International Commission on the Biological Effects of Electromagnetic Fields (ICBE-EMF), on the contrary, concluded that the thermal-based ICNIRP and FCC limits are “*invalid and continue to present a public health harm*”. The commission noted that new limits are “*urgently needed*”. They should be based on scientific results instead of “*erroneous assumptions, especially given the increasing worldwide exposures of people and the environment to RFR*” [17].

### 1.2. Evidence of Impact on Sleep

Evidence of adverse biological effects from pulsed or modulated microwave RF radiation began to appear in the scientific literature during the 1960s and 1970s. Researchers at that time reported that the central and peripheral nervous systems, including the brain, appeared to be particularly susceptible to such exposure [18,19].

Studies of occupationally exposed groups demonstrated that non-thermal levels of microwave or RF radiation were associated with a range of neurological symptoms, including sleep disturbances, fatigue, dizziness, headaches, anxiety, and difficulties with attention and memory [20,21].

Studies investigating health effects among people living close to base stations or mobile phone masts have reported increased prevalence of several of the same symptoms, including sleep problems, as those described above. There are also studies that have found no effects. Balmori [10] analyzed all studies investigating health effects among people living near mobile phone base stations between 2002 and 2021. The majority of the studies, 17 out of 23, showed increased prevalence of various symptoms described above that are due to effects on the central nervous system.

A study published in 2025 found increased prevalence of the microwave syndrome or radiofrequency sickness symptoms associated with increased measured microwave RF radiation in the investigated persons’ homes [22]. Although the levels in the highest exposure group (5000–8000 μW/m^2^) were well below the ICNIRP and the FCC limits, 91.2% of the participants in that group reported sleeping problems. Other symptoms reported by over 90% of the participants were fatigue, memory problems, headache, increased irritability, and anxiety.

In another study, the prevalence of neuropsychiatric complaints such as headache, memory changes, dizziness, tremors, depressive symptoms, and sleep disturbance was significantly higher among exposed inhabitants than controls. Memory problems and sleep disturbance were among the symptoms with the highest prevalence [23].

Statistically significant increased prevalence of headaches, dizziness, fewer hours of sleep, and tiredness were found in a study involving people living near mobile phone antennas in Vallecas, Madrid, Spain [24].

The rollout of 5G technology began around 2019/2020, accompanied by a rapid increase in the number of base stations and antennas emitting microwave RF radiation.

To date, eight individual case studies, along with a summary review covering seven of them, have been published describing health effects in persons exposed to emissions from 5G base stations [2,3,4,5,6,7,8,9,25]. The most frequent and severe symptoms were sleep disturbances followed by fatigue, headaches, and increased irritability among the 16 individuals included in these case studies [25].

In a study involving 1072 participants, those with the highest measured RF radiation levels had shorter sleep duration and longer sleep latency than those living in zones with lower measured levels [26].

A large cohort of mobile phone use and sleep disturbances with 21,049 Swedish and 3120 Finnish participants reported in 2020 a higher risk, odds ratio (OR) = 1.24, and 95% confidence interval (CI) = 1.03–1.51 of insomnia among the most intensive users (>258 min/week). The study, funded to a significant extent by telecommunication companies, found most other sleep outcomes not to be associated with mobile phone use [27].

A study involving 1925 students aged 17–23 years in Saudi Arabia found that average mobile screen use per 24 h was 8.57 ± 4.59 h in 2018/2019 and that mobile screen use of ≥8 h per day and night was related to sleep disturbances and decreased sleep time [28].

Controlled laboratory studies show strong evidence that microwave RF radiation impacts the brain’s electrical activity, measured by electroencephalogram, EEG [29]. When normal brain activity is disrupted, sleep disturbances may follow. A study involving 12 participants found that exposure to the Wi-Fi frequency 2.45 GHz for seven nights significantly reduced sleep quality [30].

A review and meta-analysis of 20 studies involving, in total, 125,198 children aged 6–19 years on use of mobile phone and portable screen-based media (tablets for instance) reported *“a strong and consistent association between bedtime media device use and inadequate sleep quantity (OR 2.17; 95% CI 1.42–3.32), poor sleep quality (OR 1.46; 95% CI 1.14–1.88), and excessive daytime sleepiness (OR 2.72; 95% CI 1.32–5.61)”* [31].

A review based on 25 studies on smart phone use and sleep quality reported that excessive smartphone use negatively impacted sleep quality and that younger populations and females were more susceptible [32].

On the other hand, a review commissioned by the WHO published in 2024 reported that microwave RF exposure below the ICNIRP limits “*does not cause symptoms, but the evidence is very uncertain. The very low certainty evidence is due the low number of studies, possible risk of bias in some studies, inconsistencies, indirectness, and imprecision*”. The review was based on five studies on sleep impact [33]. The first author, Martin Röösli, is a member of the ICNIRP, which might be a conflict of interest [34,35].

In a previous article, we described increasing numbers of memory problems among children age 5–19 years in Sweden and Norway [1]. Sleep deprivation may be related to memory problems as it has been reported to have a detrimental impact on memory [36,37].

### 1.3. Aim of This Study

The general public’s exposure to microwave RF radiation and use of mobile phones have increased substantially since the beginning of this millennium. Therefore, it is interesting to explore if sleeping problems have increased during the same time period. The aim of this study was to investigate if diagnosis of sleeping problems has increased in Sweden among various age groups, to describe trends, and to propose hypotheses.

## 2. Method

**Figure 1:**
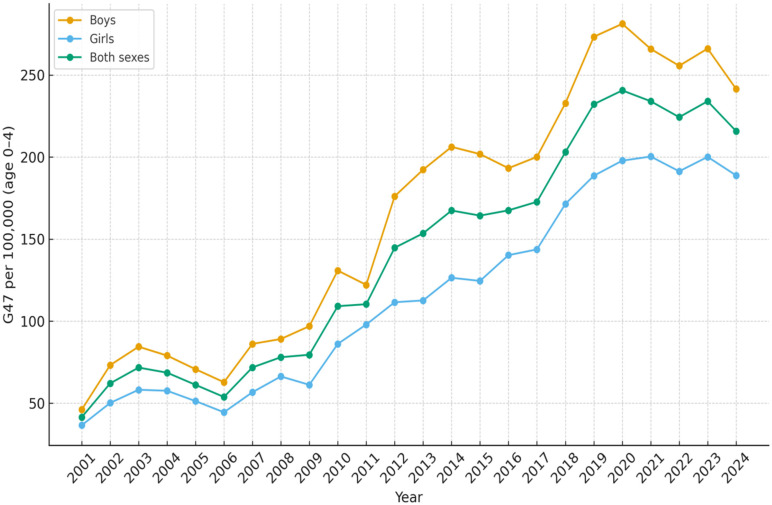
Number of patients aged 0–4 per 100,000 inhabitants diagnosed with sleep disorders (G47) as main diagnosis, each year from 2001 to 2024, in Sweden.

**Figure 2:**
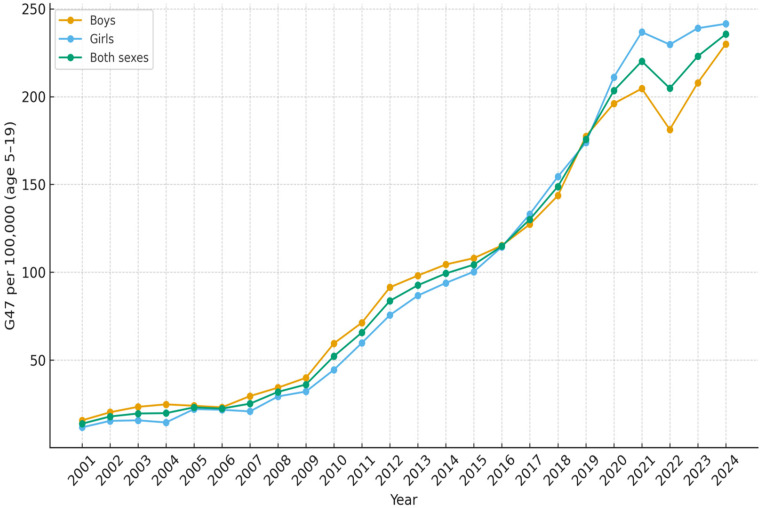
Number of patients aged 5–19 per 100,000 inhabitants diagnosed with sleep disorders (G47) as main diagnosis, each year from 2001 to 2024, in Sweden.

**Figure 3:**
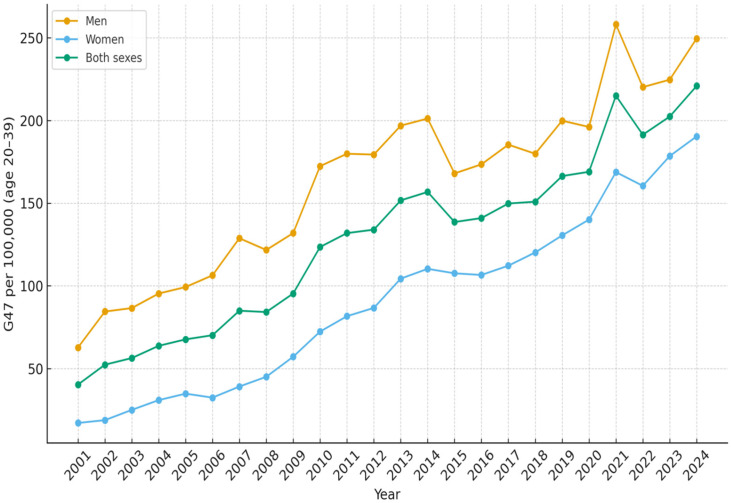
Number of patients aged 20–39 per 100,000 inhabitants diagnosed with sleep disorders (G47) as main diagnosis, each year from 2001 to 2024, in Sweden.

**Figure 4:**
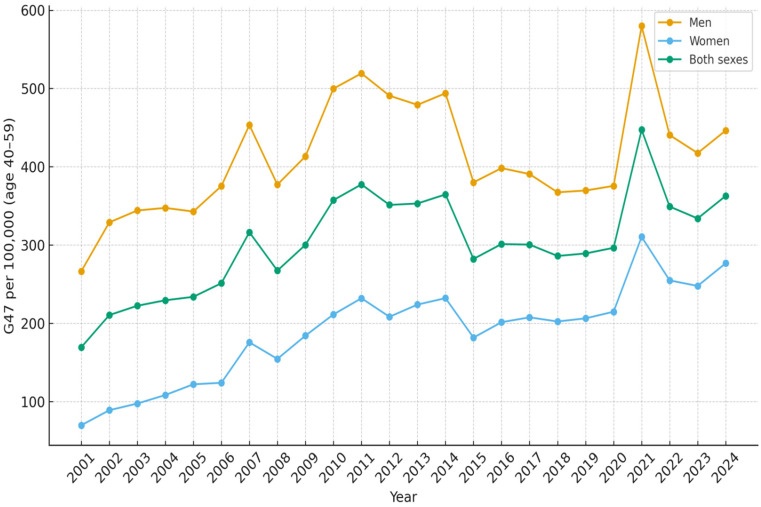
Number of patients aged 40–59 per 100,000 inhabitants diagnosed with sleep disorders (G47) as main diagnosis, each year from 2001 to 2024, in Sweden.

**Figure 5:**
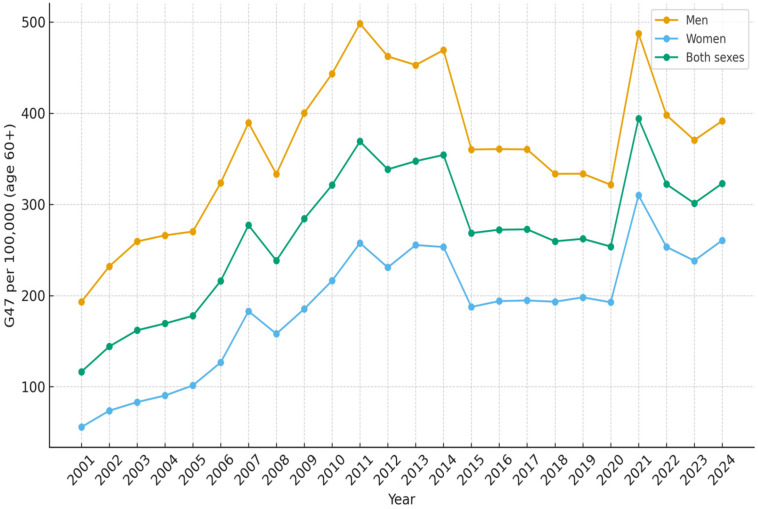
Number of patients aged 60 + per 100,000 inhabitants diagnosed with sleep disorders (G47) as main diagnosis, each year from 2001 to 2024, in Sweden.

Statistics on sleeping problems were obtained from The Swedish National Board of Health and Welfare’s national patient register on numbers of patients in specialized outpatient care diagnosed with codes for sleeping problems. We obtained numbers of patients per year and per 100,000 inhabitants in various age groups: 0–4, 5–19, 20–39, 40–59, and 60+ years diagnosed each year between 2001 and 2024 with the ICD-10 codes G47 (sleep disorders) or F51 (non-organic sleep disorders) as the main diagnosis. Since this was a register-based study without any individual data, ethics approval was not needed. AI tool Chat GPT version 5.2 was used for the making of the line charts (Figure 1, Figure 2, Figure 3, Figure 4 and Figure 5).

## 3. Results

All results are presented as the number of diagnosed patients per 100,000 inhabitants per year. In the age group 0–4 years, the number of children with sleep disorders G47 as the main diagnosis increased from 41.5 in 2001 to 215.8 in 2024 (Table 1). The increase was more rapid since 2010 and reached a peak during 2020 with 240.6 patients (Figure 1). For children and adolescents aged 5–19 years, the corresponding results were 13.8 in 2001 and 235.6 in 2024 (Table 2, Figure 2). In the age group 20–39 years, 40.4 were diagnosed in 2001 and 220.9 in 2024 (Table 3, Figure 3). The results in the age group 40–59 years were 169.5 in 2001 and 362.8 in 2024, respectively (Table 4, Figure 4). Slightly lower numbers were seen in the age group 60+ years, with 116.4 in 2001 and 322.9 in 2024 (Table 5, Figure 5).

The most pronounced increase in sleep disorders (G47) was observed in the 5–19 years age group, where the incidence per 100,000 inhabitants increased approximately 17 times between 2001 and 2024. The most rapid increase occurred after 2009, when the incidence of diagnosed sleep disorders in this age group increased from 36.1 per 100,000 inhabitants in 2009 to 235.6 per 100,000 inhabitants in 2024.

For the diagnosis F51, non-organic sleep disorders, only a small change in number of patients was observed during the same time period, and for some age groups, there was no change. The numbers were also much lower compared to G47 (data not in table).

No substantial differences were observed between males and females in the number of patients diagnosed with sleep disorders within the 5–19 years age group. There were constantly more boys than girls aged 0–4 years diagnosed with sleep disorders over the time period. In that age group, 241.5 boys and 188.8 girls per 100 000 inhabitants were diagnosed in 2024 (Figure 1). For the age groups 20 years and older, more men than women were diagnosed with sleep disturbances over the whole studied time period (Figure 3, Figure 4 and Figure 5).

## 4. Discussion

This register-based study showed increasing numbers of sleep disturbances ICD code G47 among all age groups in Sweden since 2001. The most rapid increase occurred among children, adolescents, and young adults. These age groups have historically not experienced this level of sleep disturbances, illustrated by the lower numbers during the first years of the studied period.

Many well-established factors influence sleep problems, including mental health trends, stress, socioeconomic shifts, and school stress. Pandemic effects might be a contributing factor. However, since the pandemic began in Sweden in 2020, it is unlikely to be a major factor. Since this was a register-based study without any individual data, major factors driving the increasing trend of sleep disturbances were nor possible to analyze. Diagnostic awareness and trends might be a confounding factor, but assessing them was not possible in this type of study. Another limitation is that no systematic measurements of RF radiation on a population level during the studied time period, or other potential contributing lifestyle or environmental factors with potential to influence sleep, were able to be assessed.

The data are based on the Swedish National Board of Health’s national patient register. The register was launched in 2001, and there may be some underreporting of the number of cases during the registry’s first years according to the source (see https://www.socialstyrelsen.se/statistik-och-data/statistik/statistikdatabasen/, accessed on 20 December 2025). However the most rapid increase in sleeping disorders among children 0–4 and 5–19 years was observed after 2009, which contradicts that initial underreporting would be a major cause of the steep rise.

Clearly, the results differed between G47 (sleep disorders) and F51 (non-organic sleep disorders), with increasing numbers for sleep disorders with the code G47. Non-organic sleep disorders, F51, have no clear medical cause and are primarily related to psychological or behavioral factors. G47 is used for patients who have difficulty falling asleep and who have a short night’s sleep. For further discussion, see [38].

The results are similar to a Swedish study involving 335,625 adults aged 18–74 years. Sleeping problems increased markedly between 2000 and 2016 across all age groups, although perceived work stress decreased [39].

The increasing trend of sleeping problems among children and adolescents is also mirrored in data from the Swedish National Board of Health on number of patients prescribed sleeping pills by physicians each year. In the age group 0–19 years, the number increased from 16,102 in 2011 to 112,000 in 2024, e.g., a sevenfold increase [40,41].

Increasing sleep disturbances among children and adults have also been reported from other countries. A clear increase, from 34% to 49%, in self-reported sleep problems was reported among 15–45-year-old Danes between 2010 and 2021 [42]. In Finland, an increasing trend of insomnia among children aged 11–18 was found between the mid-1990s and the end of the first decade of the 2000s [43]. A *“striking increase”* in the number of sleep problems among adults in the USA was reported between 2002 and 2012 [44,45].

Insufficient sleep has been described as a global health problem with serious health implications. Chronic insufficient sleep and sleep disorders may lead to a range of physical and mental health problems [46].

We have previously documented increasing memory problems and mild cognitive decline (including memory problems) among children and adolescents aged 5–19 in Norway and in Sweden [1]. The increase in memory problems in this age group in Sweden began after 2010, around the same time period as the more pronounced increase in sleeping problems began among children aged 0–4 and 5–19 years in Sweden based on official statistics.

The increase in sleeping problems and memory problems has coincided in time with a huge increase in the publics’ exposure to microwave RF radiation from mobile telecommunication technologies. As documented in our article on memory problems, see Table 3 in [1], children have been increasingly exposed to microwave RF radiation during the last 15 years through the introduction and marketing of smartphones, Wi-Fi in schools and homes, and the rollout of 4G and later 5G. In 2009, one of the largest telecom operators in Sweden directed a marketing campaign to parents of children aged 7 years. Prepaid mobile phone cards were offered to schoolchildren starting in the first grade [47]. By 2022, more than 70% of Swedish children aged 15 years and 40% of children aged 12 years used a mobile phone for more than 3 h per day [48]. 

Given the register-based design, this study is only hypothesis-generating and does not allow for causal inference. While the hypothesis of RF contributing to rising sleep disorder rates is biologically and socially plausible, further rigorous investigations are needed to explore possible driving factors behind the increasing sleep disturbances [46,49].

In our case studies involving a total of 16 studied persons exposed to 5G base stations near their apartment or house, the most common and severe symptom was sleeping problems, followed by fatigue [25].

Blue light from the increasing use of smartphones, tablets, and laptops may also contribute to increasing sleeping problems as the light disrupts circadian rhythm and melatonin production [50].

Sleeping problems may be caused by several factors. However, the rapid increase in recent years is clearly coinciding with a parallel increase in the public’s exposure to microwave RF radiation and increasing use of smart phones and screens. In future investigations, sleep disorders, RF radiation exposure, and biological parameters would be desirable to study.

## 5. Conclusions

This Swedish register-based study showed increasing sleeping problems in the population since about 2009, especially in the age groups up to 39 years. The increase cannot only be caused by higher prevalence of reporting patients to the national patient register. Rather, the pattern suggests the involvement of a widespread environmental- or lifestyle-related exposure. Notably, the period under study coincides with a substantial rise in population exposure to microwave RF radiation following the deployment of 4G and 5G infrastructure and the growing use of mobile phones and other RF radiation-emitting technologies. Such exposures may represent an important contributing factor to the observed trends.

## Figures and Tables

**Table 1:** Number of patients aged 0–4 per 100,000 inhabitants diagnosed with sleep disorders G47 as main diagnosis, each year from 2001 to 2024, in Sweden.

Year	Number of Patients per 100,000
2001	41.5
2002	62.1
2003	71.7
2004	68.6
2005	61.2
2006	53.8
2007	71.8
2008	78.0
2009	79.5
2010	109.1
2011	110.3
2012	144.7
2013	153.5
2014	167.4
2015	164.3
2016	167.5
2017	172.7
2018	203.0
2019	232.2
2020	240.6
2021	234.0
2022	224.3
2023	234.0
2024	215.8

**Table 2:** Number of patients aged 5–19 per 100,000 inhabitants diagnosed with sleep disorders G47 as main diagnosis, each year from 2001 to 2024, in Sweden.

Year	Number of Patients per 100,000
2001	13.8
2002	17.9
2003	19.6
2004	19.8
2005	23.1
2006	22.4
2007	25.2
2008	31.9
2009	36.1
2010	52.2
2011	65.7
2012	83.7
2013	92.6
2014	99.3
2015	104.3
2016	114.8
2017	130.1
2018	148.8
2019	175.7
2020	203.4
2021	220.2
2022	204.8
2023	223.0
2024	235.6

**Table 3:** Number of patients aged 20–39 per 100,000 inhabitants diagnosed with sleep disorders G47 as main diagnosis, each year from 2001 to 2024, in Sweden.

Year	Number of Patients per 100,000
2001	40.4
2002	52.4
2003	56.4
2004	63.8
2005	67.7
2006	70.2
2007	85.0
2008	84.2
2009	95.4
2010	123.5
2011	131.9
2012	134.0
2013	151.7
2014	156.8
2015	138.6
2016	141.0
2017	149.8
2018	150.9
2019	166.4
2020	169.0
2021	215.0
2022	191.4
2023	202.4
2024	220.9

**Table 4:** Number of patients aged 40–59 per 100,000 inhabitants diagnosed with sleep disorders G47 as main diagnosis, each year from 2001 to 2024, in Sweden.

Year	Number of Patients per 100,000
2001	169.5
2002	210.6
2003	222.5
2004	229.5
2005	233.9
2006	251.3
2007	316.4
2008	267.4
2009	300.3
2010	357.4
2011	377.5
2012	351.3
2013	353.1
2014	364.7
2015	282.3
2016	301.3
2017	300.6
2018	286.1
2019	289.3
2020	296.5
2021	447.2
2022	349.2
2023	334.0
2024	362.8

**Table 5:** Number of patients aged 60 + per 100,000 inhabitants diagnosed with sleep disorders G47 as main diagnosis, each year from 2001 to 2024, in Sweden.

Year	Number of Patients per 100,000
2001	116.4
2002	144.1
2003	161.9
2004	169.3
2005	177.7
2006	216.0
2007	277.1
2008	238.3
2009	284.2
2010	321.2
2011	369.0
2012	338.4
2013	347.4
2014	354.2
2015	268.5
2016	272.2
2017	272.6
2018	259.5
2019	262.2
2020	253.7
2021	394.1
2022	322.1
2023	301.2
2024	322.9

## Data Availability

The information generated and analyzed during the current study is available from the corresponding author on reasonable request.
